# Dicer Expression Exhibits a Tissue-Specific Diurnal Pattern That Is Lost during Aging and in Diabetes

**DOI:** 10.1371/journal.pone.0080029

**Published:** 2013-11-07

**Authors:** Yuanqing Yan, Tatiana E. Salazar, James M. Dominguez, Dung V. Nguyen, Sergio Li Calzi, Ashay D. Bhatwadekar, Xiaoping Qi, Julia V. Busik, Michael E. Boulton, Maria B. Grant

**Affiliations:** 1 Genetics and Genomics Graduate Program, University of Florida, Gainesville, Florida, United States of America; 2 Department of Pharmacology and Therapeutics, University of Florida, Gainesville, Florida, United States of America; 3 Genetics Institute, University of Florida, Gainesville, Florida, United States of America; 4 Department of Anatomy and Cell Biology, University of Florida, Gainesville, Florida, United States of America; 5 Department of Physiology, Michigan State University, East Lansing, Michigan, United States of America; University of Miami School of Medicine, United States of America

## Abstract

Dysregulation of circadian rhythmicity is identified as a key factor in disease pathogenesis. Circadian rhythmicity is controlled at both a transcriptional and post-transcriptional level suggesting the role of microRNA (miRNA) and double-stranded RNA (dsRNA) in this process. Endonuclease Dicer controls miRNA and dsRNA processing, however the role of Dicer in circadian regulation is not known. Here we demonstrate robust diurnal oscillations of Dicer expression in central and peripheral clock control systems including suprachiasmatic nucleolus (SCN), retina, liver, and bone marrow (BM). The Dicer oscillations were either reduced or phase shifted with aging and Type 2 diabetes. The decrease and phase shift of Dicer expression was associated with a similar decrease and phase shift of miRNAs 146a and 125a-5p and with an increase in toxic Alu RNA. Restoring Dicer levels and the diurnal patterns of Dicer-controlled miRNA and RNA expression may provide new therapeutic strategies for metabolic disease and aging-associated complications.

## Introduction

Most human physiological and behavioral activities, as well as cellular and metabolic functions, are under strong circadian regulation, including rest-activity cycles, body temperature rhythms, hormone secretion, and gene expression patterns[1]. In mammals, the master pacemaker, the suprachiasmatic nucleolus (SCN), is located in the hypothalamus and dictates the diurnal rhythms of the pacemakers located in peripheral tissues[2]. The circadian clock was initially modeled as interlocked transcription–translation feedback loops that drive rhythms in gene expression of core *CLOCK*, *BMAL*, *PER*, and *CRY* genes[1,3]. In addition to these classic feedback loops, circadian regulation has been demonstrated to involve posttranscriptional, translational, and posttranslational mechanisms[4,5]. Importantly microRNAs (miRNAs) and double stranded RNA (dsRNA) were shown to play a vital role in regulating various aspects of circadian clock function[4,6]. Degradation of circadian clock genes, which play an integral role in clock regulation, is controlled in part by miRNA[7].

The function of Dicer in miRNA production is well defined. Following transcription by RNA polymerase II (RNA pol II), the primary miRNA transcript (pri-miRNA) is processed by the RNase III enzyme, Drosha, into precursor-miRNA (pre-miRNA)[8]. Through interaction with exportin-5, the pre-miRNA is transported into the cytoplasm, where it is further cleaved by Dicer, resulting in the production of a mature ~22-nt RNA duplex[8,9]. One strand of the duplex is incorporated into the RNA-induced silencing complex (RISC), while the other strand is degraded. The RISC, loaded with mature miRNAs, is guided by the miRNA to pair with the target transcript and induce mRNA degradation or inhibition of translation[10]. In addition to miRNA production, Dicer is involved in long dsRNA processing independent of its RNA interference function[11].

Despite the potential importance of circadian regulation of miRNA and dsRNA production, little is known about the endogenous or physiological variation of Dicer levels and whether Dicer levels exhibit diurnal rhythm. Therefore, we sought to test whether Dicer mRNA and protein levels varied in a diurnal manner and to determine whether diurnal rhythms were tissue-specific. To determine the significance of such changes, we examined whether diurnal variations were influenced by aging and by the chronic disease state of diabetes, a condition that has been deemed “premature aging”. Our data indicate that Dicer exhibits distinct diurnal patterns in multiple tissues and that loss of diurnal rhythmicity and phase shifts of Dicer are associated with aging and with diabetes.

## Results

### Tissue-specific diurnal patterns of Dicer expression

**Figure 1:**
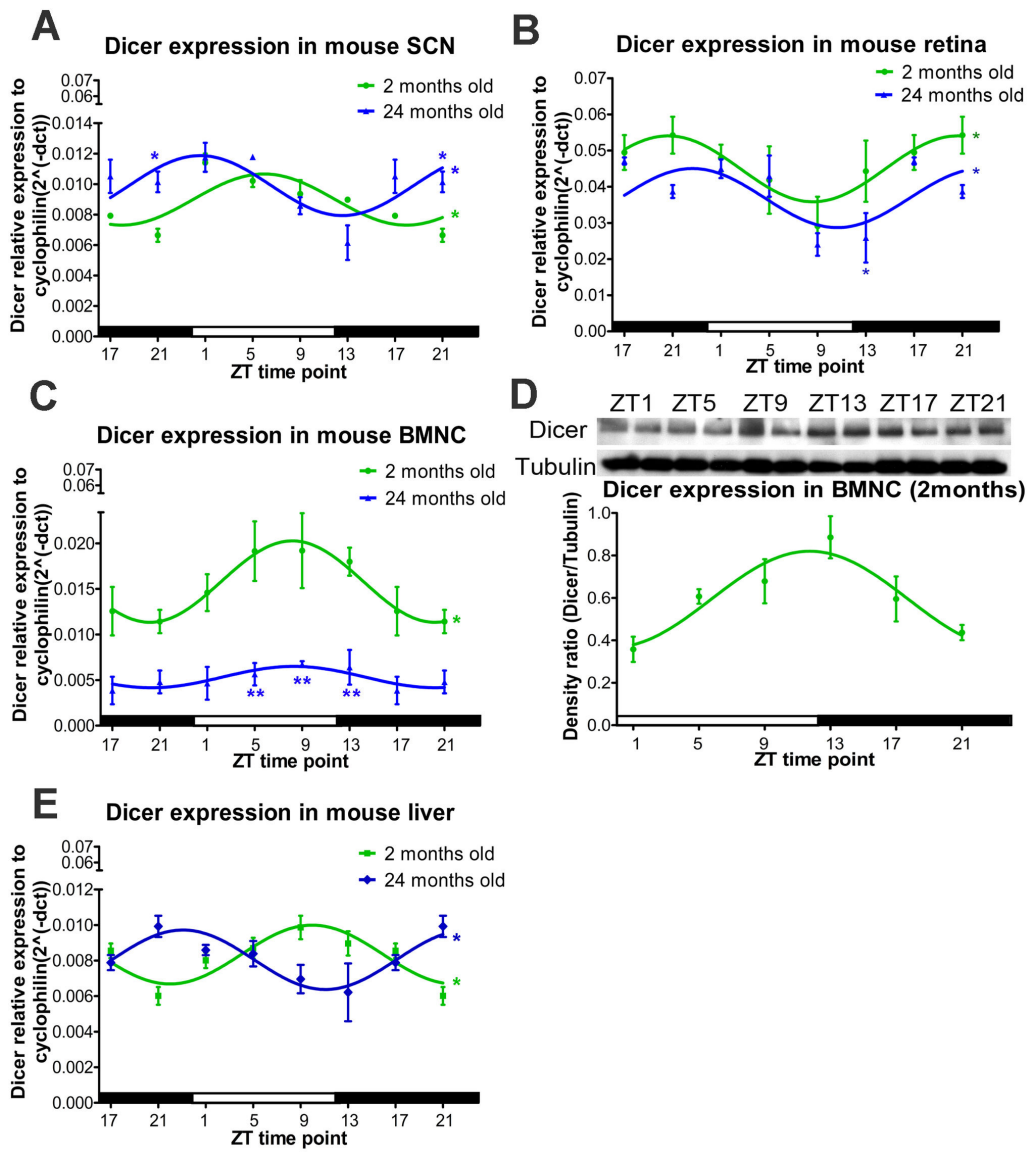
Diurnal Dicer expression is altered in tissues from aged mice. (A) Dicer mRNA expression in mouse SCN shows diurnal oscillation with a phase advance observed in 24-month-old mice compared to 2-month-old mice. (B) Diurnal oscillation of Dicer expression occurs in the mouse retina, but the overall expression was reduced in aged mice. (C,D) In mouse BMNC, diurnal oscillation of Dicer mRNA (C) and protein expression (D) is lost in 24-month-old mice. (E) The oscillation patterns observed in livers of 2- and 24-month-old mice are in antiphase. For RNA expression, n=4 at each ZT time point for 2-month-old mice, and n=3 for 24-month-old mice. In (A)-(C) and (E), time points 17 and 21 have been duplicated to facilitate viewing of the time curve. The significance of diurnal oscillation by zero-amplitude test was indicated in the right end of the smooth curve. The significant difference at each time point between different age groups was indicated either above or below the SEM indicator.

Clock gene expression varies with time of day and tissue origin. Certain tissues, such as the liver, exhibit high amplitudes of *PER1* and *PER3*, while lower amplitudes are observed in the SCN and retina[12]. To evaluate whether Dicer exhibited a similar diurnal tissue-specific pattern, the levels of Dicer mRNA were examined in the SCN, liver, retina, and bone marrow mononuclear cells (BMNC) from young mice. Diurnal variation was observed in all four tissues; however, the peak of expression varied between tissues. In the SCN and BMNC, peak expression was observed at Zeitgeber Time (ZT) 5. In the retina (light-entrained), peak expression was observed at ZT21, while peak expression was observed at ZT9 in liver (food-entrained; Figure 1 green line).

### Reduction and phase shift of Dicer expression in aged mice

In the aging individual, diurnal oscillations are altered with a phase-advanced sleep-wake cycle, fragmented sleep, shortened circadian period length, and dampened circadian amplitude[1,13,14]. With aging, the ability of the SCN to entrain the circadian system to local time is reduced, and this difference leads to the loss of temporal coordination and defective homeostatic regulation, rendering individuals more vulnerable to disease[14,15]. To evaluate whether the diurnal pattern of Dicer mRNA expression was altered with age, we compared the response in 2-month-old mice to that in 24-month-old mice. While Dicer expression exhibited clear diurnal variation in the SCN of young mice (with a peak at ZT5), a phase advance with a peak at ZT1 was observed in 24-month-old mice (Figure 1A). In the feeding-entrained liver, Dicer mRNA was in antiphase in the aged mice compared with the young mice (Figure 1E). In the BMNC (Figure 1C), young mice exhibited peak Dicer expression at ZT5, while the expression level in aged mice was markedly reduced for all the time points. In the retina, a light-entrained tissue, the aged mice exhibited reduced Dicer expression level, most notably at ZT13 (Figure 1B). These distinct patterns of Dicer mRNA expression indicate tissue-specific changes in expression as well as clear age-related phase changes or amplitude reductions.

### Reduced Dicer expression in diabetic retina

**Figure 2:**
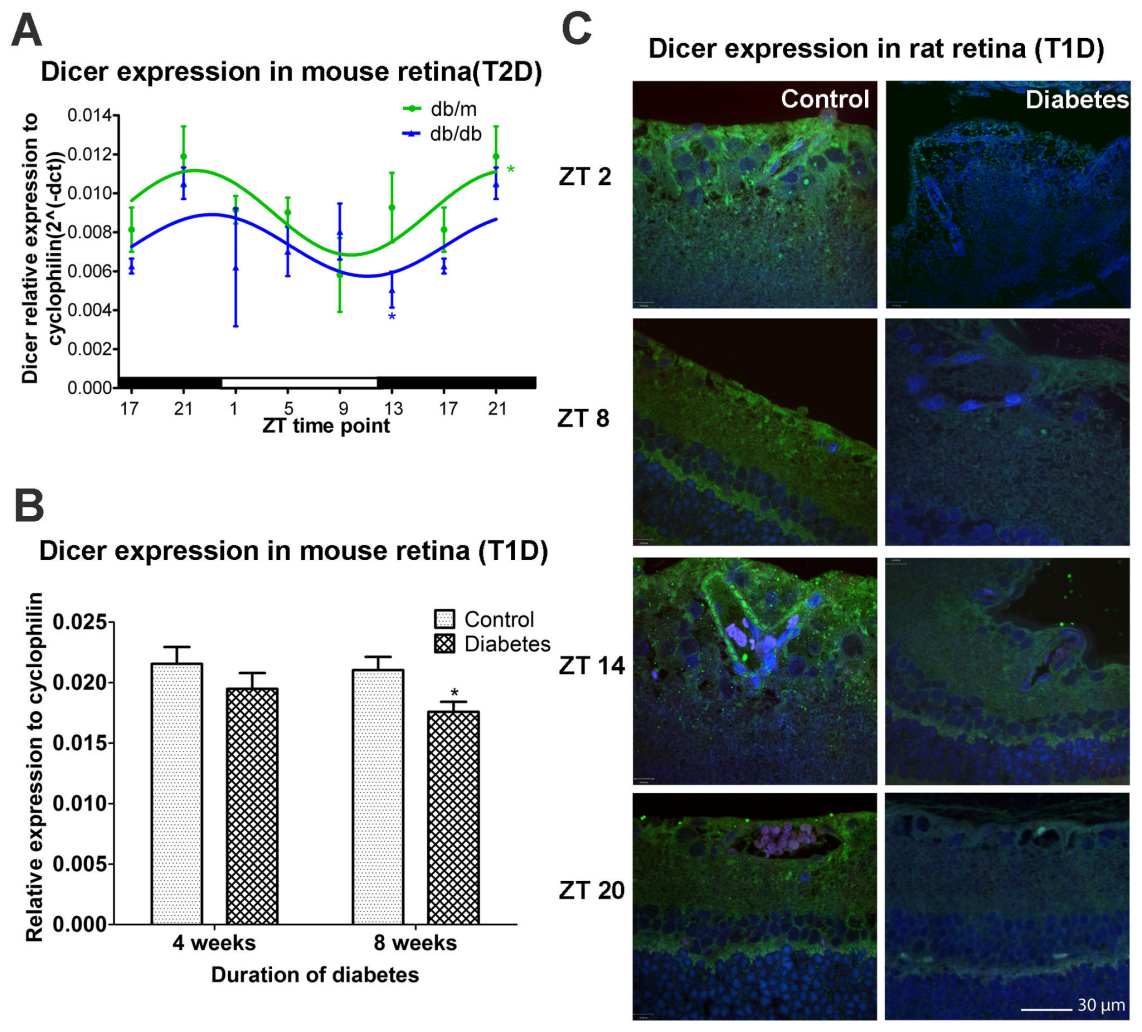
Reduced Dicer expression in diabetic retinas. (A) Control (db/m, n=3) and T2D (db/db, n=3) mice were euthanized at each time point (4-h intervals) for 24 h. Dicer mRNA expression from control mouse retina showed diurnal oscillation with the peak observed at ZT21 and the nadir at ZT9. Dicer expression was lower in T2D mice. (B) STZ-induced T1D mice with 4 and 8 weeks diabetes duration were euthanized at ZT9 (nadir of Dicer expression). Dicer mRNA expression was not significantly different at 4 weeks of diabetes mice, but was reduced at 8 weeks of diabetes. (C) In control rats, the retina exhibits a diurnal pattern of Dicer protein expression with peaks observed at ZT2 and ZT14; however, in T1D rat retinas, Dicer protein expression was decreased. In (A), Time points 17 and 21 have been duplicated to facilitate viewing of the time curve. For T2D mice, n=3 at each ZT time point for each group. The mRNA expression in T1D mouse (n=6) is shown for each ZT time point for each group.

Much like aging, diabetes is associated with circadian disruption[16,17]. We demonstrated that both type 1 diabetes (T1D) and T2D are associated with loss of rhythmicity of progenitor cells released from the BM and that this loss promotes vascular dysfunction due to insufficient vascular repair and development of diabetic retinopathy[17]. Diabetes is considered a state of premature aging, largely due to the similarity of the vascular pathology in both situations[18]. Thus, we examined the expression of Dicer mRNA in the 11 month T2D db/db mouse and age-matched controls. As observed for the young wild-type mice, peak Dicer expression in the retina occurred at ZT21 in the control db/m mice (Figure 2A). In the db/db mice, the expression level was reduced in the retinas at ZT13, as observed in the aged mice (Figure 2A). To explore these findings in a T1D mouse model, we examined streptozotocin (STZ)-induced diabetic mice at 4 and 8 weeks of diabetes. At ZT9, which represented the lowest Dicer mRNA expression in the retina (Figure 2B), diabetic mice at 8 weeks of diabetes, but not at 4 weeks of diabetes, showed reduced Dicer mRNA expression when compared with that in age-matched non-diabetic control mice (Figure 2B). These results indicate that Dicer mRNA is reduced in the retinas in both T1D and T2D murine models of diabetes.

Dicer expression was decreased in aged and diabetic mouse retina. To further test whether this decreased expression occurred in other species, STZ-induced diabetic rat retinas were examined. Dicer proteins were abundantly detected throughout the normal rat retina (Figure 2C). The protein was expressed at the highest levels at ZT2 and ZT14, while the expression levels were lowest at ZT8 and ZT20 in normal eyes (Figure 2C). However, the staining was diminished in eyes collected from 4-month-old T1D rats (Figure 2C), which was consistent with the changes we observed for Dicer expression in diabetic mouse retinas (Figure 2A,B).

### Reduced Dicer expression in diabetic mouse bone marrow progenitors cells

**Figure 3:**
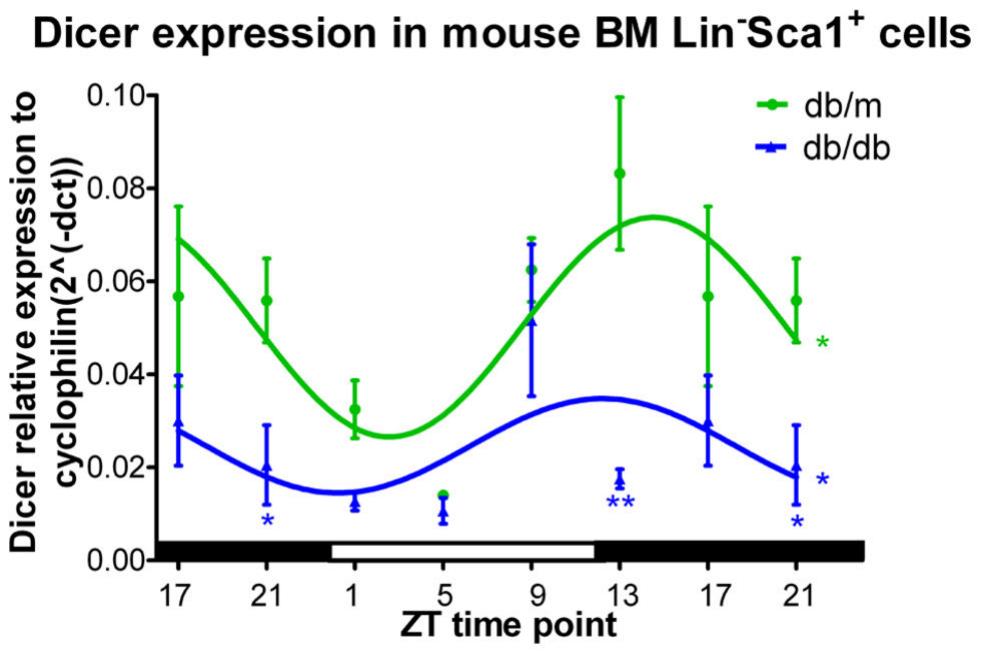
BM Lin^-^Sca1^+^ cells obtained from the bone marrow of T2D mice, euthanized every 4 hrs, showed reduced Dicer mRNA expression. BM Lin^-^Sca1^+^ cells from 11-month-old T2D (db/db) mice were isolated, and RT-PCR was performed to quantify Dicer expression. Diurnal oscillation of Dicer expression in bone marrow Lin^-^Sca1^+^ cells was observed in both control and diabetic mice. The expression level was greatly reduced in diabetic animals. Time points 17 and 21 have been duplicated to facilitate viewing of the time curve. N=3 at each ZT time point for each group.

Diabetic retinopathy results partly from the loss of adequate endothelial repair due to loss of progenitor cell function[19]. To further examine Dicer expression in T2D mice, 11 month old (db/db) mice were sacrificed every 4 hours and bone marrow progenitor cells (i.e., Lin^-^Sca1^+^ cells) were isolated and gene expression levels were quantified. In the progenitor cells of db/m (control) mice, Dicer exhibited a robust diurnal expression pattern with a peak at ZT13 and a nadir at ZT1 (Figure 3). In 11-month-old db/db mice, the diurnal expression pattern was maintained in bone marrow Lin^-^Sca1^+^ cells (Figure 3); however, the expression level was decreased, especially at the peak time point (Figure 3).

### Reduced Dicer expression in progenitor populations of diabetic human subjects

**Figure 4:**
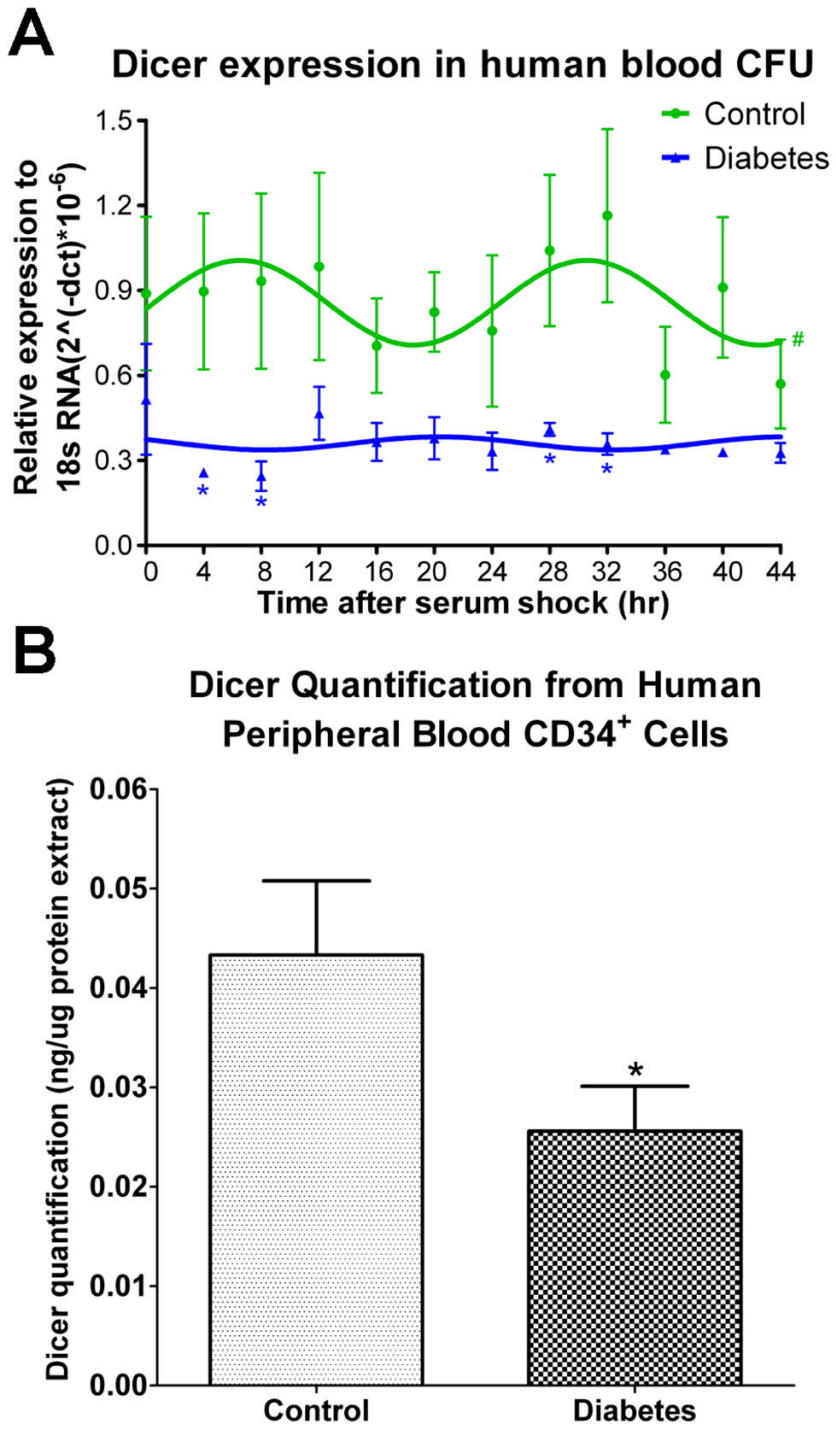
Dicer expression is reduced in progenitor cells from T2D human subjects. (A) Human peripheral blood was seeded in 35-mm dishes for 14 days to observe colony formation. Cells were maintained in serum-free medium following a 50% serum shock for 2 h. Cells were isolated every 4 h for mRNA expression. Dicer expression from healthy human peripheral blood colonies showed diurnal oscillation with peaks observed 8 and 32 h following serum shock. Loss of diurnal oscillation and reduced mRNA expression of Dicer was observed in colonies fromT2D subjects. (B) Dicer expression in freshly isolated diabetic CD34^+^ cells was reduced compared to age- and sex-matched controls.

 Previously, we showed that human retinal endothelial cells (HRECs) and CD34^+^ cells isolated from diabetic donors exhibit “diabetic memory” *in vitro* and maintain dysfunction[20]. Thus, we examined two different human progenitor populations, colony-forming unit-endothelial cells (CFU-ECs) and peripheral blood CD34^+^ cells, from T2D and age- and sex-matched human subjects. As shown in Figure 4, CFU-ECs from healthy individuals retained diurnal oscillations (Figure 4A) following serum shock to synchronize the cells. In contrast, the CFU-ECs derived from diabetic subjects showed lower Dicer mRNA expression (Figure 4A). In addition, Dicer protein expression was lower in freshly isolated CD34^+^ cells from T2D subjects than from control subjects (Figure 4B).

### Decrease and phase shift of miRNAs 146a and 125a-5p in aged mice

**Figure 5:**
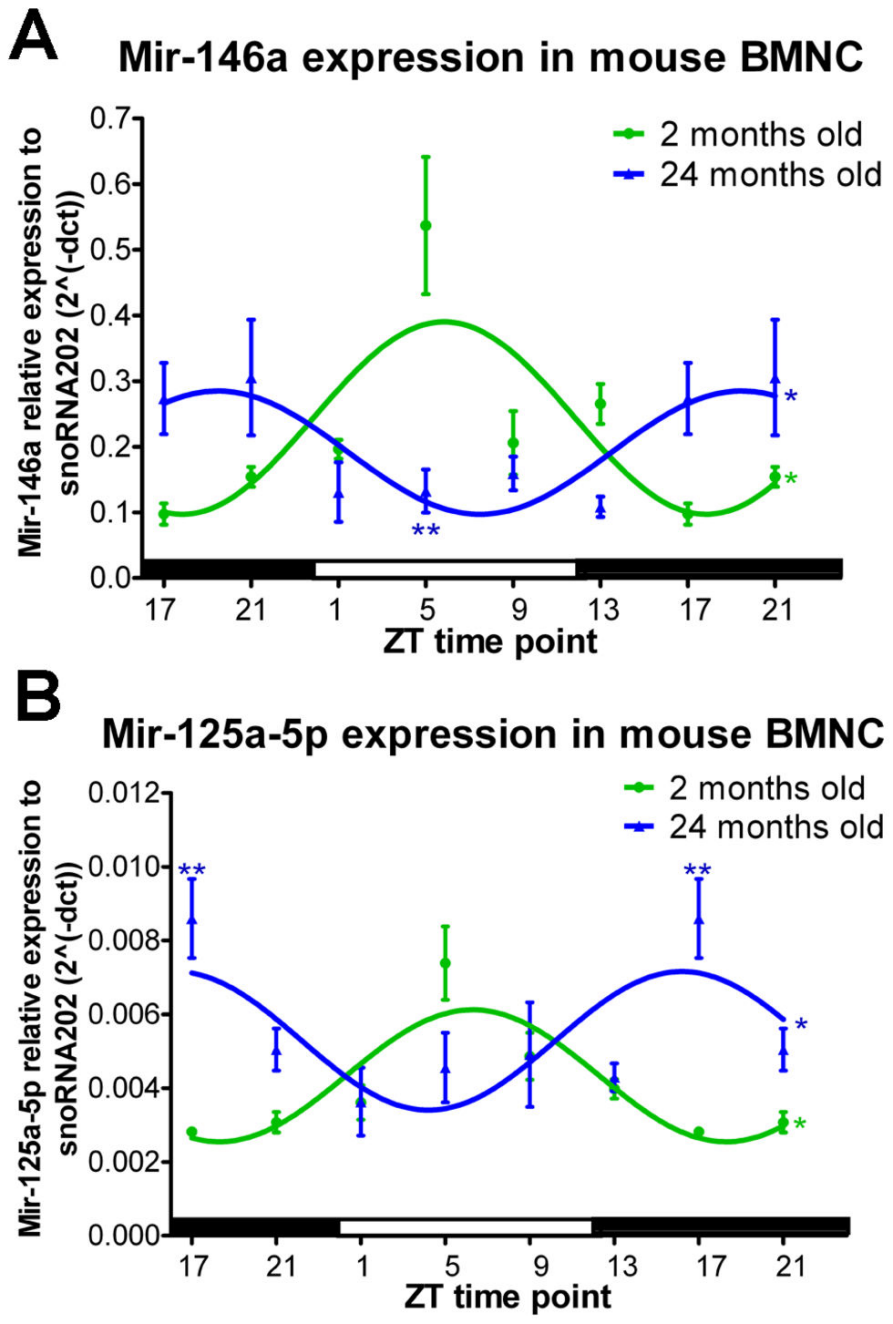
Reduced miRNA expression in BMNC from aged mice. (A) Expression of miR-146a showed a diurnal pattern in both young and aged mice; however, the expression pattern was phase delayed and reduced in diabetic mice. (B) Diurnal oscillation of miR-125a-5p with a phase delay was observed in diabetic animals. Time points 17 and 21 have been duplicated to facilitate viewing of the time curve. N=4 for 2-month-old mice, and n=3 for 24-month-old mice at each ZT time point.

One of the essential roles of Dicer is to process the maturation of miRNAs. The diurnal expression of Dicer in BMNC from young mice coincided with the diurnal expression pattern of miR-146a, a miRNA that is responsible for the pro-inflammatory reaction[21], and of miR-125a-5p, which is involved in the cell apoptosis pathway (Figure 5A,B)[22]. Due to the reduced levels and loss of diurnal oscillation of Dicer expression in BMNC from aged mice, the diurnal patterns of miR-146a and miR-125a-5p were phase delayed, and the expression of miR-146a was reduced at ZT 5(Figure 5A,B).

### Increased plasma Alu RNA in T2D human subjects

**Figure 6:**
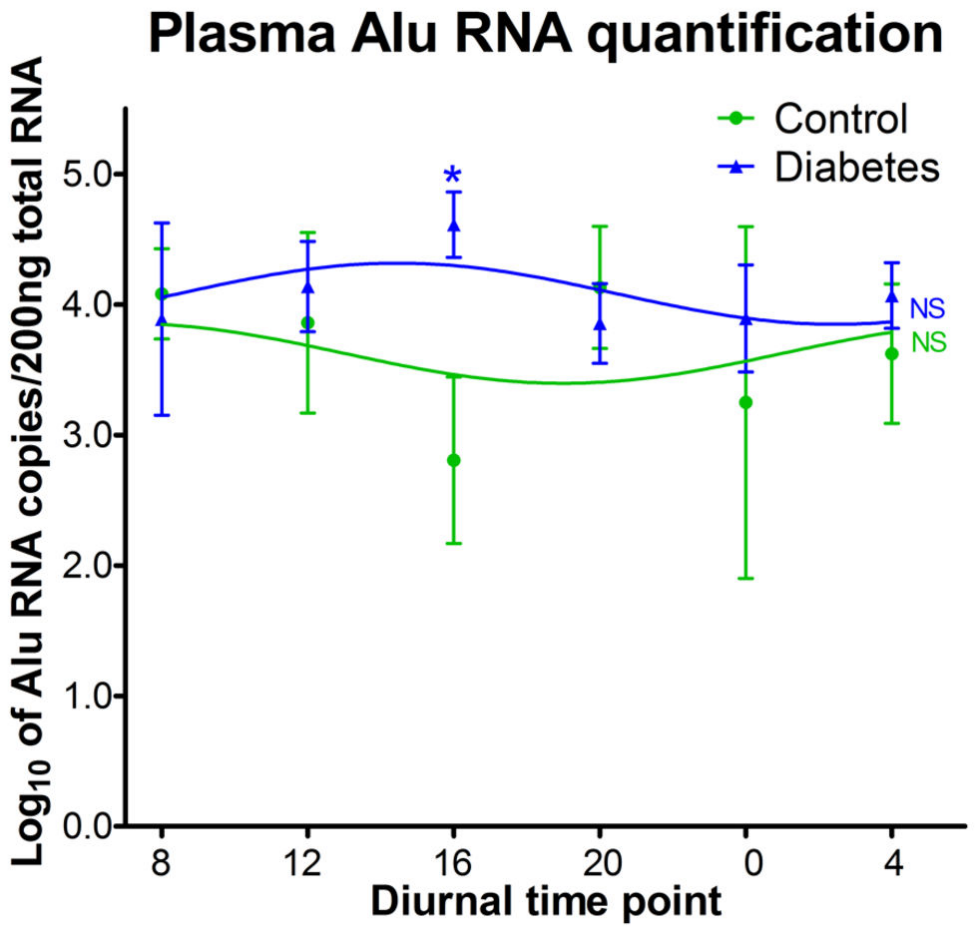
T2D human subjects show an increase in *Alu* RNA expression in plasma. To quantify *Alu* RNA, control (n=4) and T2D subjects (n=7) underwent plasma collection at 4-h intervals. No diurnal oscillation of *Alu* RNA expression was observed in control or diabetic human plasma. The expression level of *Alu* RNA in diabetic subjects was significantly higher than that in controls at 4pm.

To determine whether reduced Dicer expression leads to increased Alu RNA in diabetes, diabetic individuals and age- and sex-matched controls were examined over a 24-h period. Blood sampling occurred every 4 h, and the diurnal pattern of Alu RNA expression in plasma was measured. No diurnal oscillation of Alu RNA expression was observed in control or diabetic human plasma. As compared to non-diabetic subjects, diabetic subjects demonstrated an increase in Alu RNA in plasma (Figure 6).

### Toxicity of Alu RNA in human retinal endothelial cells (HRECs) and bone marrow Lin^-^ *Sca1* ^+^ cells

**Figure 7:**
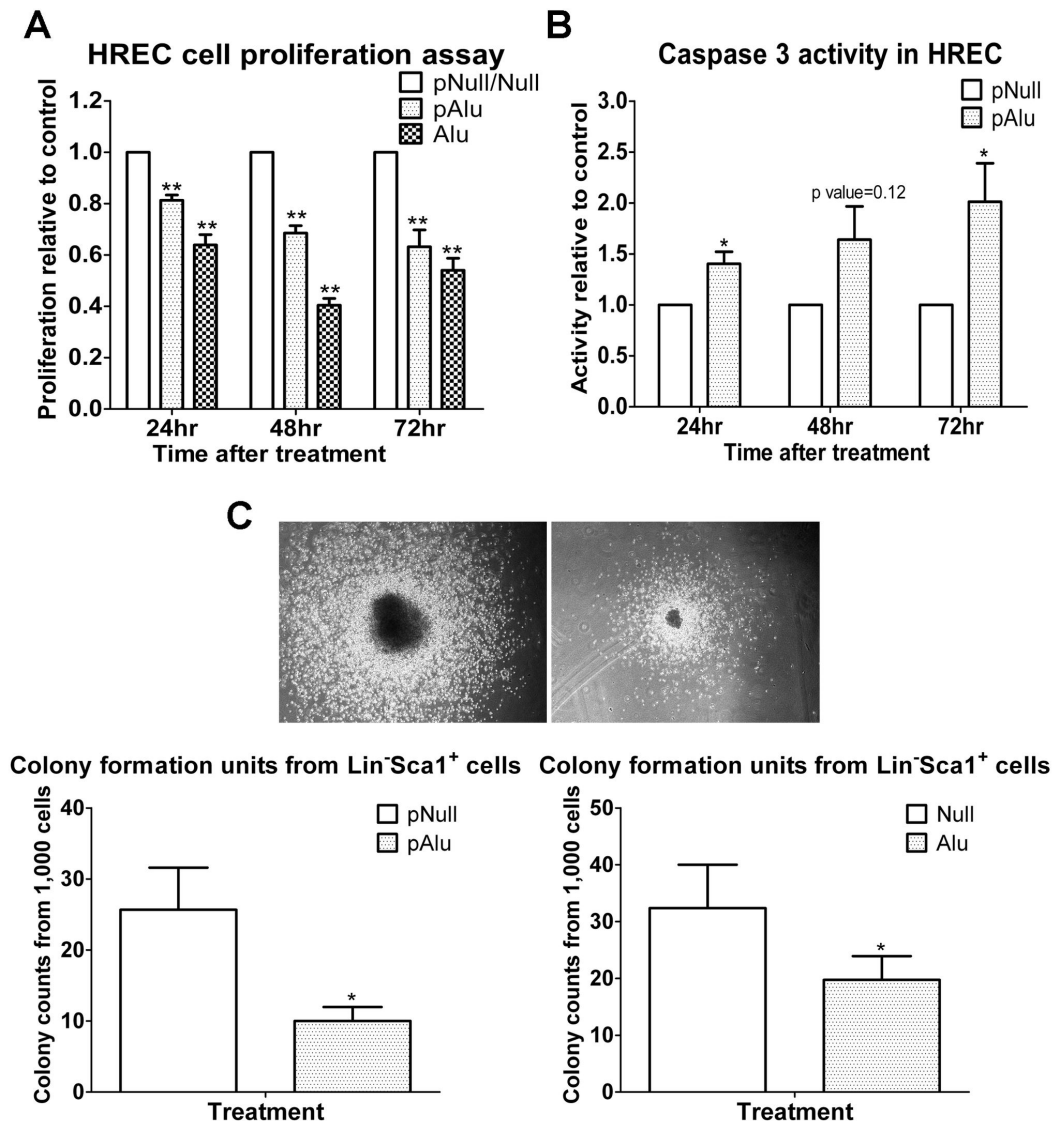
Alu RNA overexpression results in HREC and BM Lin^-^Sca1^+^ cell toxicity. (A-C) HRECs and BM-derived Lin^-^Sca1^+^ cells were transfected with plasmid expressing Alu or Alu RNA. (A,B) In HRECs, cell proliferation and caspase 3 activity were examined 24, 48, and 72 h post-transfection. (A) Alu RNA reduced HREC proliferation and (B) increased caspase 3 activity. (C) BM-derived Lin^-^Sca1^+^ cells were seeded in culture for colony formation analysis post-transfection. Alu RNA over expression reduced bone marrow Lin^-^Sca1^+^ cell colony formation.

Dicer is a key component of the RNA interference pathway and is critical for removal of toxic Alu RNA from cells[11]. Increased levels of Alu RNA in retinal pigment epithelial (RPE) cells can result in cell death[11]. Therefore, we examined whether increased expression of Alu RNA resulted in toxicity in HRECs and murine BM Lin^-^Sca1^+^ cells. HRECs were transfected with either control plasmid, Alu-expressing plasmid, or Alu RNA. The cells transfected with either the Alu plasmid or the Alu RNA showed reduced cell proliferation (Figure 7A), which was most marked at 48 h post-transfection. Previously, Alu toxicity was shown to result in increased caspase 3 activation[11] in RPE cells. Thus, we determined whether transfecting cells with Alu-expressing plasmid would have a similar effect on HRECs. Indeed, HRECs exposed to the Alu-expressing plasmid demonstrated increased caspase 3 activity (Figure 7B). Similarly, a colony formation assay demonstrated that overexpression of Alu RNA in BM Lin^-^Sca1^+^ cells resulted in a decrease in the number and size of colonies formed (Figure 7C).

## Discussion

Dicer is an RNase III family endoribonuclease that plays an important role in dsRNA and pre-miRNA processing by cleaving these molecules into short dsRNA fragments to produce miRNA and siRNA. With a growing understanding of the important role for miRNA and siRNA in mammalian physiology, it is not surprising that Dicer is essential for normal development [23-28] and for regulation of various physiological and pathological processes, including fertility[29] and neural[30], thyroid[31], cardiac[28], hepatic[32], pancreatic[33], and kidney[34-36] cell function.

 Most physiological processes are known to be under both central and peripheral circadian control. The circadian clock is comprised of transcriptional/translational feedback loops involving the clock genes. Clock genes, in turn, regulate hormonal secretion and metabolism in accordance with the environmental light–dark cycle through direct effects on a myriad of clock-controlled genes. There is an emerging appreciation of the role of circadian dysfunction, sleep disturbances and metabolic disease in particular in the pathogenesis of T2D[37]. Disrupted circadian rhythms caused by disturbed sleep-wake cycles, inactivity during the active period, enhanced activity during the rest period and high food consumption might lead to attenuated feeding rhythms, disrupted metabolism and obesity[38]. These lifestyles with disrupted circadian physiology may result in high parasympathetic output to viscera leading to obesity, hyperinsulinemia, and hyperlipidemia, or high sympathetic output to the muscle and heart leading to vasoconstriction and hypertension[39]. Furthermore, findings in murine models show a strong link between genetic background and circadian rhythm disruption in determining the severity of metabolic disorders[38].

Recent studies demonstrated that miRNAs are intricately involved in the circadian regulation of clock genes[4,6,40] and clock-controlled genes. In turn, miRNA expression is controlled by clock genes[41,42]. Our group and others have previously shown that diabetes[17], metabolic syndrome[43], and aging[44,45] are associated with dysregulation of both the central and peripheral circadian clock. Using the T2D rat model, we demonstrated that downregulation of circadian genes in the retina, SCN, and bone marrow is associated with the development of diabetic retinopathy[17]. Moreover, release of vascular progenitors from the bone marrow was highly synchronized to the vascular repair process with a peak at ZT5 in control animals, although the peak of vascular progenitor release was diminished in diabetic animals[17,46].

Several studies demonstrated an important role of miRNA in metabolic regulation[47] and aging[48,49]. Dicer is one of the rate-limiting enzymes in miRNA processing. In this study, we determined whether the observed circadian miRNA patterns are due in part, to circadian variation in Dicer expression. We found a robust circadian pattern for Dicer expression in all the analyzed tissues (SCN, retina, BMNC, and liver). Dicer exhibited differential circadian patterns in these distinct tissues with the highest expression and the strongest oscillations in the retina. These findings correlate with published data showing that circadian patterns of miRNA expression are highly tissue-dependent and that the retina has a large number of miRNAs that exhibit a circadian expression pattern[50,51]. Aging and diabetes led to a phase shift of Dicer expression in the SCN and liver and an overall decrease in Dicer expression in retina and BMNC. These changes were accompanied by a reduction and a phase shift of miR-146a and miR-125a in the BMNC.

In addition to miRNA, Dicer has an important function in dsRNA processing. A reduction of Dicer-1 in RPE cells has been shown to lead to severe macular degeneration through dsRNA accumulation, especially Alu RNA accumulation[11]. In agreement with the Dicer reduction data, diabetes led to an increase in plasma Alu RNA expression at 4 PM , a time associated with the circadian reductions of serum cortisol[52]. Moreover, treatment of HRECs and BM Lin^-^Sca1^+^ cells with Alu RNA decreased cell proliferation and colony formation, respectively and led to an increase in caspase 3 activity, which supports that both progenitor populations and mature cells are vulnerable to the toxicity of Alu RNA. 

 In summary, this study demonstrates the circadian regulation of Dicer, an important endoribonuclease from the RNase III family, in multiple organs. Aging and diabetes led to either the loss of the circadian pattern of Dicer or the downregulation of Dicer expression, and these effects were reflected in the circadian patterns of both types of important RNAs controlled by Dicer, miRNAs and toxic Alu RNA. Therefore, restoring Dicer levels and the diurnal patterns of Dicer-controlled miRNA and dsRNA expression could provide new therapeutic strategies for the correction of metabolic disease and aging-associated organ damage.

## Materials and Methods

### Animals

All animal procedures were in agreement with the NIH Guide for the Care and Use of Laboratory Animals, the ARVO Statement for the Use of Animals in Ophthalmic and Vision Research, and institutional guidelines. All studies were approved by the University of Florida Institutional Animal Care and Use Committee. Male C57BL/6 mice of two and 24 months of age were used for the studies described in Figure 1. C57BL/6 and B6.BKS(D)-Leprdb/J (stock number 000697) mice were obtained from the Jackson Laboratory (Bar Harbor, ME) and housed in the animal care facilities at the University of Florida with a strict 12h:12h light:dark cycle. STZ-induced T1D animals were generated as previously described[53]. Bones, retinas, livers, and brains were harvested every 4 h. Animals were euthanized and dissected under red light during the dark period. Eyes and brains were maintained in RNAlater (Life technologies, Grand Island, NY) at 4°C until they were processed. Livers were snap frozen in liquid nitrogen and stored at -80°C until analysis. Bone marrow cavities were flushed, and then cells were removed, snap frozen, and stored at -80°C.

### Cell culture

HRECs were grown in complete medium (DMEM/Hams F12 with 10% fetal bovine serum [FBS], 5 μg/mL transferrin, 2 μg/mL selenium, 1 μg/mL insulin, 0.584 mg/mL glutamine, and 15 mg/mL endothelial growth supplement (Sigma-Aldrich, St. Louis, MO) at 37°C in a humidified 5% CO_2_ atmosphere. Cells were used for experiments at passage 4-6. Lipofectamine™ 2000 (Life technologies, Grand Island, NY) was used to transfect the Alu plasmid and dsRNAs into HRECs. Both Alu plasmid and dsRNA were provided by Dr J. Ambati, University of Kentucky.

### Isolation of bone marrow cells

The murine equivalent of the human CD34^+^ cell is the Lin^-^Sca1^+^ cell. To isolate mouse Lin^-^Sca1^+^ cells, whole BM cells were flushed into PBS with 2% FBS followed by lysis of red blood cells with ammonium chloride (Stemcell Technologies, Vancouver, Canada). Lineage cells were depleted using a commercial kit (cat#:19756; Stemcell Technologies, Vancouver, Canada). Lin^-^Sca1^+^ cells were enriched by positive selection from lineage-negative cells (cat#:18756; Stemcell Technologies, Vancouver, Canada).

### Quantitative RT-PCR

Dicer and cyclophilin mRNA levels were examined in mouse tissues. MiRNeasy mini kit (Qiagen, Valencia, CA) was used to extract total RNA, and this RNA was then used to synthesize cDNA by iScript^TM^ cDNA Synthesis Kit (Biorad, Pleasanton, CA) for mRNA analysis or converted into cDNA by TaqMan MicroRNA Reverse Transcription Kit (Life technologies, Grand Island, NY) for miRNA analysis. Using commercial primers (Dicer:Mm00521722_m1; cyclophilin:Mm02342430_g1; Life technologies, Grand Island, NY), cDNA levels were determined using cyclophilin as an internal control. Quantitative real-time PCR was carried out using the Applied Biosystems 700 Fast Real-time PCR system (Life technologies, Grand Island, NY). To quantify miR-146a, miR-125a-5p, and snoRNA202, the miRNA-specific primers (miR-146a: 000468; miR-125a-5p:002198; snoRNA202: 001232; Life technologies, Grand Island, NY) were used for real-time PCR using TaqMan® Universal PCR Master Mix ( Life technologies, Grand Island, NY). To quantify human plasma Alu RNA, total RNA from plasma was extracted using the Trizol LS reagent (Life technologies, Grand Island, NY). The RNA was converted into cDNA using the QuantiTect Reverse Transcription Kit (Qiagen, Valencia, CA). The primer set for Alu RNA (FP: 5′-CAACATAGTGAAACCCCGTCTCT-3′; RP: 5′-GCCTCAGCCTCCCGAGTAG-3′) was synthesized by Integrated DNA Technologies(IDT, San Diego, CA). Absolute quantification of Alu RNA in plasma was performed using SYBR® Green Real-Time PCR Master Mixes (Life technologies, Grand Island, NY) with the standard for Alu RNA kindly provided by Dr J Ambati.

### Western blotting

Cell pellets or tissues were lysed via incubation with RIPA buffer and a protease inhibitor cocktail (Sigma-Aldrich, St. Louis, MO) on ice. Protein concentration was measured with the BCA Protein Assay following the manufacturer’s instructions (Biorad, Pleasanton, CA). Anti-Dicer (Abcam,Cambridge, MA) and anti-tubulin (Cell Signaling Technology, Boston, MA) monoclonal antibodies were used to probe the target protein.

### Immunofluorescence microscopy

Eyes were removed from STZ-induced T1D rats that had a 4-month duration of diabetes and from age-matched controls. Eyes were processed for standard paraffin embedding, and 4-µm sections were cut and air-dried. Sections were then deparaffinized/rehydrated in xylene and serial concentrations of ethanol. Antigen unmasking was done with Rodent Declocker (Catalog# RD913L; Biocare Medical, Concord, CA), and then the sections were blocked with 10% normal goat sera and 5% BSA for 1 h at room temperature. Mouse monoclonal anti-Dicer (Abcam,Cambridge, MA) antibodies were diluted in PBS with 1% normal goat sera plus 1% BSA and incubated with the samples for 2 h at room temperature. After washing with PBS, the sections were incubated with secondary antibodies conjugated with FITC for 1 h at room temperature in the dark. Sections were covered with Vectashield mounting medium/DAPI (Vector Laboratories , Inc. Burlingame, CA). Photographs were taken using a Zeiss Florescent microscope.

### Human studies

The study was approved by the University of Florida IRB #411-2010. All study subjects provided informed consent. Patients were brought into the Clinical Research Center at the University of Florida for 48 h. During the first 24 h, patients were evaluated and on the evening of the first day, a heparin lock was inserted into their forearm. During the second 24-h period, the patients had 1 mL of blood removed every 4 h for a total of 24 h, and this blood was used for analysis of Alu RNA. For the second study, diabetic (n=10) and non-diabetic patients (n=10) were examined, and 150 mL of blood was drawn to isolate CD34^+^cells.

Patient Characteristics: Subjects were diagnosed with T2D. Baseline characteristics, including current age, diabetes duration, glycosylated hemoglobin level (HbA1c), lipid parameters, body mass index, and blood pressure, were recorded. Controls were deemed healthy and matched to the diabetic subjects for age and sex.

Inclusion criteria: Individuals between the ages of 21 and 65 years were eligible to participate.

Exclusion criteria: Subjects were excluded for the following reasons: a) evidence of ongoing acute or chronic infection (HIV, Hepatitis B or C, or tuberculosis); b) ongoing malignancy; c) cerebral vascular accident or cerebral vascular procedure; d) current pregnancy; e) history of organ transplantation; f) presence of a graft; g) uremic symptoms, such as an estimated glomerular filtration rate of less than 20 cc/min (by Modification of Diet in Renal Disease equation), or an albumin of less than 3.6 (to avoid malnutrition as a confounding variable); h) a history of smoking; and i) anemia.

### CD34^+^ cell isolation

CD34^+^ cells were isolated from blood, and the mononuclear cells were harvested using Ficoll-Paque plus solution (GE healthcare, Milwaukee, WI). Red blood cells were lysed in the mononuclear cell preparation using ammonium chloride (Stemcell Technologies, Vancouver, Canada). CD34^+^ cells were enriched by EasySep™ Human CD34 Positive Selection Kit according to manufacturer’s instructions (Stemcell Technologies Vancouver, Canada).

### Murine CFU-EC isolation

To obtain progenitor cell colonies, peripheral blood was drawn from control and diabetic mice. Red blood cells were lysed with ammonium chloride (Stemcell Technologies Vancouver, Canada), and mononuclear cells were mixed with Methocult medium (Stemcell Technologies, Vancouver, Canada) and incubated at 37°C in a humidified chamber with 5% CO_2_ atmosphere for 14-16 days. Colonies were harvested in Stemspan medium (Stemcell Technologies, Vancouver, Canada) and then subjected to 50% horse serum shock before collection at 4-h intervals for 44 h.

### Dicer ELISA quantification

Protein lysates were extracted from the CD34^+^ cell pellet in cold PBS by ultrasonication. The concentration of the CD34^+^ protein lysate was measured by the Bradford method (Biorad, Pleasanton, CA). Protein lysate (500 ng) was loaded into wells of a 96-well plate and incubated with a series of reagents according to the product instructions of Ribonuclease Type III (DICER1) ELISA Kit (USCN Life Science, Hubei, China). 

### Murine CFU-EC analysis

Murine bone marrow Lin^-^Sca^+^ cells were enriched by magnetic selection (Stemcell Technologies, Vancouver, Canada). Lin^-^Sca^+^ cells (1×10^3^) were transfected with Alu RNA, plasmid expressing Alu, or control plasmid for 8 h and then seeded for colony formation analysis. Colonies were enumerated after 10 days in culture.

### Cell proliferation assay

HRECs were transfected with Alu RNA, a plasmid expressing Alu, or control plasmid for a period of 24, 48, or 72 h. CellTiter 96® AQueous One Solution Reagent (20 μL) (Promega, Madison, WI) was added to each well of the 96-well assay plate. The plate was incubated at 37°C for 2 h in a humidified chamber with 5% CO_2_ atmosphere. The reaction was blocked by addition of 10% SDS (30 μL) into each well. The absorbance was recorded at 490 nm using a 96-well plate reader. The proliferation ratio was calculated based on the absorbance.

### Caspase 3 activity assay

HRECs were transfected with either the control plasmid, the plasmid expressing Alu, or Alu RNA for 24, 48, or 72 h. Cell pellets were collected, and cold lysis buffer was added. The protein concentration was measured using the BCA Protein Assay (Biorad, Pleasanton, CA). Reaction buffer and caspase 3 fluorogenic substrate (DEVD-AFC) were added, and the mixture was incubated at 37°C for 2 h (R&D Systems, Minneapolis, MI). Light emission was measured at a wavelength of 505 nm using filters that allow light excitation at a wavelength of 400 nm.

### Statistical analysis

The data was plotted as mean ± SEM. The diurnal data represents time series data, and single cosine analysis was employed to fit the data into a smooth curve. The data was considered diurnal oscillation by the zero-amplitude test with a *p*-value of less than 0.05. The expression level between different age groups was compared using two-way ANOVA with Tukey post hoc analyses (GraphPad Prism, San Diego, CA). Asterisk indicates statistical significance at *p* ≤ 0.05, and two asterisks indicate significance at *p* ≤ 0.01.
